# The Philadelphia Pathological Society

**Published:** 1878-11

**Authors:** 


### THE PHILADELPHIA PATHOLOGICAL SOCIETY.

---

PHILADELPHIA, Thursday, September 26, 1878.

The President, Dr. H. Lenox Hodge, in the chair.

Dr. John H. Brinton opened the discussion by exhibiting specimens from six fatal cases of stricture of the urethra.

Case I. A man, thirty-seven years of age, in whose case the parts had first been stretched, and internal urethrotomy then performed. A well-marked attack of urethral fever followed the introduction of the catheter several days after the operation, accompanied by vomiting and suppression of urine. The patient died on the fifth day. The autopsy showed the kidneys to be large and congested; an old, false passage running from a point one inch in front of, to a point two inches behind, the stricture; great congestion of the prostatic sinus, in the near neighborhood of which there was an almond-sized cavity filled with pus; bladder slightly congested; neighboring veins in a highly phlebitic condition; phlebitis the evident cause of death.

Case II. Internal urethrotomy performed, followed by introduction of No. 10 Thompson sound. Death on the forty-third day; bladder hypertrophied and contracted, scarcely holding two fluid ounces of urine; stricture entirely healed; death caused by multiple abscesses of both kidneys.

Case III. Stricture dilated gradually; no operation performed. Death on thirty-seventh day; bladder full of pus; false passage; death caused by severe pyelitis.

Case IV. Stricture dilated; no operation performed. Kidneys dilated and congested; bladder contracted and lined with tubercular deposits; large urinary abscess reaching nearly to the fundus of the bladder; all urine passed through a fistula; death on the fifth day, caused by inflammation of the lungs.

Case V. Stricture dilated by Holt's instrument. Kidneys full of multilocular cysts. Death on forty-seventh day, due to this cystic disease of kidneys.

Case VI. Meatus cut and stricture dilated at three o'clock in the afternoon; surgical fever entirely controlled by opium and quinine. The man turned over in bed, and died without a groan at six o'clock on the following morning.

In commenting upon the above cases, Dr. Brinton said that all the deaths had occurred in his hospital practice; that he had never lost a single case in private practice; that he had been able, by carefully tabulating all his cases, to reach the same conclusion drawn by Sir Henry Thompson, namely, that two-thirds of all strictures are to be found in the bulbo-membranous portion of the urethra; that in a total of sixty-eight cases of stricture following gonorrhœa, the average period elapsing between the time of gonorrhœal infection and the appearance of the stricture had been found to be from five to seven years. The doctor further stated that urethral fever appeared in three out of a total of seven cases in which divulsion and urethrotomy had been performed in all of the cases in which internal urethrotomy had been employed, namely, four; in eight out of a total of twelve cases where divulsion after Holt's method had been used; in all of the cases of perineal section, namely, four; and in eight out of a total of twenty-two cases where gradual dilatation was employed.

Dr. Samuel W. Gross had paid much attention lately to the subject under discussion, and wished to call attention to the fact that in cases of stricture where other portions of the genito-urinary apparatus were involved, any operation, no matter how slight it might be, in hospital practice was likely to be followed by death; that patients entering the hospitals with venereal diseases and stricture were, as a usual thing, entirely broken down in constitution before applying for medical aid; that in such cases he had known death to follow the passage of a catheter. He wished to call attention further to the fact that only the most gentle methods of treatment had been employed in Dr. Brinton's cases, and that what seemed to be a very ghastly showing was due simply to the causes which he had just pointed out.

In his case, as in Dr. Brinton's, the results of private practice had been altogether different, but of twenty-five hospital cases he had three deaths following internal urethrotomy, and one death following divulsion; whereas in a total of seventy-six cases in private practice, in forty-seven of which he had performed internal urethrotomy, and in twenty-nine divulsion, he had not lost a single case. He said that he always gave a full dose of quinia before attempting any operation on the urethra, and after the operation injected a good quantity of morphia under the skin. He stated that of the four fatal cases in his hospital practice one death had been caused by pyæmia, and in the other three cases a *post-mortem* examination had shown the kidneys to be granular and contracted; that no albumen or tube casts had been found in the urine of these patients.

Dr. Gross's results differed somewhat from those reached by Dr. Brinton and Sir Henry Thompson; in only about forty-six per cent. of his cases had the stricture been located in the bulbo-membranous portion of the urethra. He desired to lay particular stress upon the following conclusions which had forced themselves upon him, namely: first, that when a stricture is found in the glandular portion of the urethra there will almost always be another further back; second, that protracted gonorrhœa was the most frequent cause of stricture; third, that divulsion and perineal section were the worst of all the operations for the relief of stricture; and, fourth, that in his experience most of the cases of urethral fever were found to follow the operation for internal urethrotomy.

In closing, Dr. Gross wished to state as his positive conviction that gradual dilatation was only a palliative means of treatment, and that a stricture could never be permanently cured except by cutting it; that before performing internal urethrotomy, however, he always made it a rule to put his patient through a course of what might be called gradual dilatation.

Dr. Charles W. Hunter wished to know whether urethral fever occurred after the passage of the catheter, as some authorities held.

Dr. Brinton replied that he placed no confidence upon such statements; that in his practice urethral fever had occurred irrespective of catheterism altogether, in some cases before, and in others after, the introduction of the catheter.

Dr. Hunter wished to know if stricture had ever reappeared in Dr. Gross's experience after internal urethrotomy.

Dr. Gross said that it had been impossible to follow up most of his cases, but that in those few which he had kept in view there had been no return of the stricture.

Dr. O'Hara desired to inquire as to the means of determining whether a fever was urethral or not.

Dr. Brinton, in answer, detailed the symptoms of urethral fever—the chill, the profuse sweating, the rapid pulse, etc.,—and said that if fever appeared five or six hours after the operation it was generally of purely nervous origin; if twenty-four hours after the operation it was in all probability urethral fever; and if as late as seventy-two hours after operating it was, without doubt, pyæmic in nature.

Dr. Charles B. Nancrede related a case in his practice in which a complete cure had followed internal urethrotomy. Dr. Brinton also mentioned several cases.

Dr. Hunter had never employed any method for the cure of stricture but that by gradual dilatation at the University Hospital, and had never as yet known of a single case permanently cured. He wished to inquire into the rationale of internal urethrotomy. Why was it that cutting the stricture did any good? He thought that the tendency of all cicatrices was to contract steadily. He certainly knew this to be so in the case of burns of the skin.

Dr. Gross was aware that cicatrices contracted steadily as a general rule, but said this was not the case in the operation for division of a stricture, and for this reason he never allowed the sides of the wound to heal together, but forced them to heal by granulation by the daily introduction of bougies of large calibre thoroughly distending the parts. That in this way instead of a constantly contracting cicatrix he was able to add a new fold of mucous membrane to the calibre of the stricture—a fold equal in length to the combined width of the sides of the cut.

Dr. Edward O. Shakespeare had quite recently, in company with Drs. D. Hayes Agnew and I. Henry C. Simes, examined under the microscope sections from several cicatrices following the operation of internal urethrotomy, and in every instance had found a large preponderance of elastic fibres in the stricture of the cicatrix; in one instance as much as seventy-five per cent. of its bulk being composed of elastic fibres. It was thus plain that cicatrices of the mucous membrane of the urethra were different from those of the skin, since there was always a preponderance of fibrous tissue in the latter.

Dr. I. H. C. Simes confirmed Dr. Shakespeare in the above statement of the results of his investgation.

Dr. Charles B. Nancrede called the attention of the society to the extreme value of this discovery, and said that Sir Henry Thompson in his most recent publications had denied the existence of elastic tissue in urethral cicatrices.

The president, Dr. H. Lenox Hodge, wished to dwell upon one fact which he thought had been overlooked in the discussion, namely, that proper significance had not been laid upon the almost universal and necessary implication of other organs in stricture of the urethra as an element always of possible fatality in the diagnosis, not only in hospital cases, but also in private practice. Dr. Hodge could remember one case in his own practice in which entire cure had followed gradual dilatation of a stricture.

Dr. Carl Seiler thought that there was always less contraction in a cicatrix of the mucous membranes of the body than in one of the epidermis.

Dr. Hunter, in opposition to this statement, instanced a case of a child who swallowed sulphuric acid, the calibre of whose œsophagus had been entirely obliterated, causing death by starvation.

Dr. Shakespeare was sorry that he could not agree with Dr. Seiler's statement.—*Boston Med. and Surg. Journal.*

